# Periodic fevers associated with celiac disease and marked increase of NK cells

**DOI:** 10.1186/1546-0096-13-S1-P192

**Published:** 2015-09-28

**Authors:** A Sediva

**Affiliations:** 1 Motol University Hospital, 2nd Faculty of Medicine, Department of Immunology, Prague, Czech Republic

## Case report

A five year old boy was referred to our department for a history of repeated fevers. Family history was uneventful, however his father suffered from repeated episodes of tonsilitis in childhood.

Our patient presented with a history of repeated fevers with a clinical signs of tonsilitis since he was 2.5 years old. Fever attacks recur approximately every 6 weeks and are associated with lymphadenitis, sometimes accompanied by aphthous stomatitis. His inflammatory markers were elevated in a time of fever episode, but did not reach very high values typical for periodic fever syndromes. Chronic fatigue and progressive anemia also formed a persistent part of his clinical presentation.

Immunological investigation revealed significant decrease of T lymphocytes (CD3+ cells between 25-40%, normal values above 60%, absolute number between 0.5-0.9x10/9/l, normal values above 1x10/9/l), and a surprising increase of NK cells reaching over 50% of lymphocyte population. Further detailed search for underlying cause led to an unexpected diagnosis of celiac disease. Appropriate diet was introduced and led promptly to a decrease of celiac disease associated antibodies and to an improvement of anemia and fatigue.

His fever attacks, however, did not resolve, and his highly pathological findings of low T cells and extremely high NK cells were still detectable, not responding to antiinflammatory and corticosteroid treatment. PET scan was performed and showed an accumulation of activity in a pharyngeal arch and tonsil area. Tonsilectomy was performed at his 6 years of age and led to a prompt improvement in his clinical presentation, with a complete resolution of febrile episodes. His abnormal laboratory findings in cellular immune parameters very slowly improved within the next 6 months, with an increase of CD3+ T cells and drop of NK cells to their normal values.

## Conclusion

Here we present an unusual combination of periodic fevers with celiac disease, accompanied by peculiar and highly significant changes in cellular immunity, with low T cells and high NK cells. Combination of celiac diet and tonsillectomy led to a resolution of all clinical problems. Slow and gradual improvement of deep immune disbalance in lymphocyte subpopulations took further 6 months after tonsillectomy before it reached almost normal values. Association of celiac disease and periodic fevers is extremely rarely mentioned in a literature, but might be considered in some cases. Aphthous stomatitis is a common feature of both celiac disease and PFAPA syndrome.

## Consent to publish

Written informated consent for publication of their clinical details was obtained from the patient/parent/guardian/relative of the patient.

